# Supplementary material for: Global HIV prevention, treatment, and care interventions and strategies for key populations: Protocol for a scoping review
Source: PLoS One. 2025 Nov 20;20(11):e0337075. doi: 10.1371/journal.pone.0337075 (PMC12633940; doi:10.1371/journal.pone.0337075)
Supplement: S3 Appendix — (DOCX) [file pone.0337075.s003.docx]

| **S3 Appendix: Data extraction tool for the scoping review** | | | | |  |  | |  |  | |  |
| --- | --- | --- | --- | --- | --- | --- | --- | --- | --- | --- | --- |
| Author(s), Year of publication | Study setting (country) | Study objective | Study design | Sample characteristics | Intervention/Strategy | Reported outcomes | Factors influencing effectiveness | | | Barriers to implementation | |
|  |  |  |  |  |  |  |  | | |  | |
|  |  |  |  |  |  |  |  | | |  | |
|  |  |  |  |  |  |  |  | | |  | |
|  |  |  |  |  |  |  |  | | |  | |
|  |  |  |  |  |  |  | |  |  | |  |
