# Supplementary material for: Global HIV prevention, treatment, and care interventions and strategies for key populations: Protocol for a scoping review
Source: PLoS One. 2025 Nov 20;20(11):e0337075. doi: 10.1371/journal.pone.0337075 (PMC12633940; doi:10.1371/journal.pone.0337075)
Supplement: Attachment — Submitted filename: Clara Oguji Comments_PLOS ONE .docx [file pone.0337075.s004.docx]

**Comments: Clara Oguji- HIV Prevention, Treatment, and Care Interventions and Strategies for Key Populations: A Systematic Scoping Review Protocol**

This is a clear, timely, and policy-relevant review protocol that addresses important gaps in HIV services for key populations. The rationale is strong, and using the JBI method adds rigor, while the inclusion of grey literature and plans to develop an integrated framework strengthen its relevance. However, some parts of the manuscript would benefit from clearer explanations, updating timelines and rephrasing.

| Title | The topic is relevant and important; however, it could benefit from revision:   1. Consider including a geographical focus; is this for the global community or Africa, or Europe?   2. Consider emphasizing the outcome of the review, “ A scoping review of interventions for key populations, rather than a Protocol.   3. This way we can clarify the “Why”. |
| --- | --- |
| Abstract Introduction | Consider specifying who the key populations are early in the abstract/introduction for clarity. This will help readers immediately understand the target groups of interest. |
| Line 35, 36 | Consider specifying who the key populations are early in the abstract/introduction for clarity. This will help readers immediately understand the target groups of interest. |
| Line 61, 62 | This is good evidence. Can you consider the proportion of this 88 million that are key population? If available? |
| Line 81- 84 | I would suggest you move this up. I was waiting to see the definition of the Key population. |
| Line 123-124 | Consider briefly justifying the choice of a scoping review over a systematic review. While the broad scope and numerous evidence sources align with this approach, you should explicitly state why a scoping review is more appropriate. |
| Line 132 | Consider combining objectives 4 and 5 to avoid redundancy. Except you want the framework to be a standalone objective |
| Line 161 -163 | Are we considering any location for this? As mentioned in my previous comment. |
| Line 167 | Consider Rephrasing to “ We will search PubMed to identify……. |
| Line 272 | Kindly review the timeline. |
| Line 278 -279 | In addition, it could limit the ability to make definitive practice recommendations. |
| Line 384 | Double-checked for formatting, and kindly add the date assessed. |
